# Ethnoracial Disparities in SARS-CoV-2 Seroprevalence in a Large Cohort of Individuals in Central North Carolina from April to December 2020

**DOI:** 10.1128/msphere.00841-21

**Published:** 2022-05-19

**Authors:** Cesar A. Lopez, Clark H. Cunningham, Sierra Pugh, Katerina Brandt, Usaphea P. Vanna, Matthew J. Delacruz, Quique Guerra, D. Ryan Bhowmik, Samuel J. Goldstein, Yixuan J. Hou, Margaret Gearhart, Christine Wiethorn, Candace Pope, Carolyn Amditis, Kathryn Pruitt, Cinthia Newberry-Dillon, John L. Schmitz, Lakshmanane Premkumar, Adaora A. Adimora, Ralph S. Baric, Michael Emch, Ross M. Boyce, Allison E. Aiello, Bailey K. Fosdick, Daniel B. Larremore, Aravinda M. de Silva, Jonathan J. Juliano, Alena J. Markmann

**Affiliations:** a Department of Microbiology and Immunology, University of North Carolina School of Medicine, Chapel Hill, North Carolina, USA; b Department of Genetics, School of Medicine, University of North Carolina at Chapel Hill, Chapel Hill, North Carolina, USA; c Department of Statistics, Colorado State University, Fort Collins, Colorado, USA; d Department of Geography, University of North Carolina at Chapel Hill, Chapel Hill, North Carolina, USA; e Carolina Population Center, Chapel Hill, North Carolina, USA; f Department of Environmental Sciences and Engineering, The University of North Carolina at Chapel Hill, Chapel Hill, North Carolina, USA; g Department of Epidemiology, School of Public Health, University of North Carolina at Chapel Hill, Chapel Hill, North Carolina, USA; h McLendon Clinical Laboratories, UNC Healthcare, Chapel Hill, North Carolina, USA; i Johnston Health Laboratories, Johnston Health, Smithfield, North Carolina, USA; j Rex Healthcare Laboratory, UNC Healthcare, Raleigh, North Carolina, USA; k Chatham Clinical Laboratory, Chatham Hospital, Siler City, North Carolina, USA; l Department of Pathology & Laboratory Medicine, University of North Carolina School of Medicine, Chapel Hill, North Carolina, USA; m Department of Medicine, Division of Infectious Diseases, University of North Carolina School of Medicine, Chapel Hill, North Carolina, USA; n Department of Computer Science, University of Colorado Boulder, Boulder, Colorado, USA; o BioFrontiers Institute, University of Colorado Boulder, Boulder, Colorado, USA; Mount Sinai School of Medicine

**Keywords:** COVID-19, SARS-CoV-2, health disparities, neutralization, seroprevalence

## Abstract

Severe acute respiratory syndrome coronavirus 2 (SARS-CoV-2) has caused millions of deaths around the world within the past 2 years. Transmission within the United States has been heterogeneously distributed by geography and social factors with little data from North Carolina. Here, we describe results from a weekly cross-sectional study of 12,471 unique hospital remnant samples from 19 April to 26 December 2020 collected by four clinical sites within the University of North Carolina Health system, with a majority of samples from urban, outpatient populations in central North Carolina. We employed a Bayesian inference model to calculate SARS-CoV-2 spike protein immunoglobulin prevalence estimates and conditional odds ratios for seropositivity. Furthermore, we analyzed a subset of these seropositive samples for neutralizing antibodies. We observed an increase in seroprevalence from 2.9 (95% confidence interval [CI], 1.8 to 4.5) to 12.8 (95% CI, 10.6 to 15.2) over the course of the study. Latinx individuals had the highest odds ratio of SARS-CoV-2 exposure at 6.56 (95% CI, 4.66 to 9.44). Our findings aid in quantifying the degree of asymmetric SARS-CoV-2 exposure by ethnoracial grouping. We also find that 49% of a subset of seropositive individuals had detectable neutralizing antibodies, which was skewed toward those with recent respiratory infection symptoms.

**IMPORTANCE** PCR-confirmed SARS-CoV-2 cases underestimate true prevalence. Few robust community-level SARS-CoV-2 ethnoracial and overall prevalence estimates have been published for North Carolina in 2020. Mortality has been concentrated among ethnoracial minorities and may result from a high likelihood of SARS-CoV-2 exposure, which we observe was particularly high among Latinx individuals in North Carolina. Additionally, neutralizing antibody titers are a known correlate of protection. Our observation that development of SARS-CoV-2 neutralizing antibodies may be inconsistent and dependent on severity of symptoms makes vaccination a high priority despite prior exposure.

## INTRODUCTION

Severe acute respiratory syndrome coronavirus 2 (SARS-CoV-2) has caused over 200 million infections and over 5 million deaths as of November 2021 due to the respiratory disease it causes, COVID-19 (1). Serological testing can be deployed efficiently at the population level (2) and complements molecular testing for evaluating the spread of SARS-CoV-2, especially given the high rates of reported asymptomatic cases as well as poor access to molecular testing. Large prevalence studies in 2020 using remnant samples from health care settings in the United States reported geographic variation in prevalence—around 30% in New York but less than 3% in North Carolina (NC), the focus of the present study (3–5). Notably, two studies overlap our cohort both temporally and geographically. One study of asymptomatic inpatients and outpatients in central NC from 28 April to 19 June 2020 found an estimated SARS-CoV-2 seroprevalence of 0.7 to 0.8% over time, and another study of remnant clinical samples (3,817 from NC) from 27 July to 24 September 2020 found an estimated seroprevalence of 2.5 to 6.8% over this time period (6, 7). Studies from other regions of the American South have also revealed variation in seroprevalence by demographic factors as well as self-reported disease (8–10). While overall seroprevalence estimates of a given study depend on sampling method, assay characteristics, geography, and temporal factors, seroprevalence studies provide information on the spread of COVID-19 that is missed by looking at the number of confirmed acute cases alone. These same cohorts can be leveraged to study the development of functionally neutralizing protective antibodies in cohorts of patients without biasing toward studying only those of severe symptoms. It is suspected that mild COVID-19 may result in lower to undetectable levels of neutralizing antibody titers (11, 12).

Seroprevalence studies are also useful for identifying social factors such as racial, ethnic, and socioeconomic disparities that may leave some to be more commonly exposed to SARS-CoV-2 (4, 10, 13). The COVID-19 pandemic has been shaped by the deep and historic impacts of structural racism on disease disparities in U.S. society as identified by serologic studies as well as hospitalization and mortality rates (14, 15). For example, COVID-19 case and hospitalization rates among black, Hispanic, and Native American populations in the United States, according to the Centers for Disease Control and Prevention, are 2.5 to 4.5 times higher than those in white populations (16). Structural and occupational factors previously identified as drivers of race and ethnic disparities in health include unequal labor market opportunities and higher representation in essential work positions that lack job security, access to infection prevention control, benefits, and sick leave (17–22). In North Carolina, non-Latinx black and Latinx individuals had significantly higher diagnostic test positivity by PCR (23).

In this study, we aim to expand on existing literature of disparate SARS-CoV-2 exposure among racial and ethnic groups in the United States by measuring seroprevalence in a large southern U.S. health care-seeking cohort in North Carolina using remnant blood samples. We also measure functionally neutralizing antibodies from a subset of these samples in order to understand the rates of detectable protective neutralizing antibodies in a prevalence cohort that includes those who had mild and asymptomatic infection, which we expect to be lower than in cohorts of symptomatic infection.

## RESULTS

### Study population characteristics.

**FIG 1:**
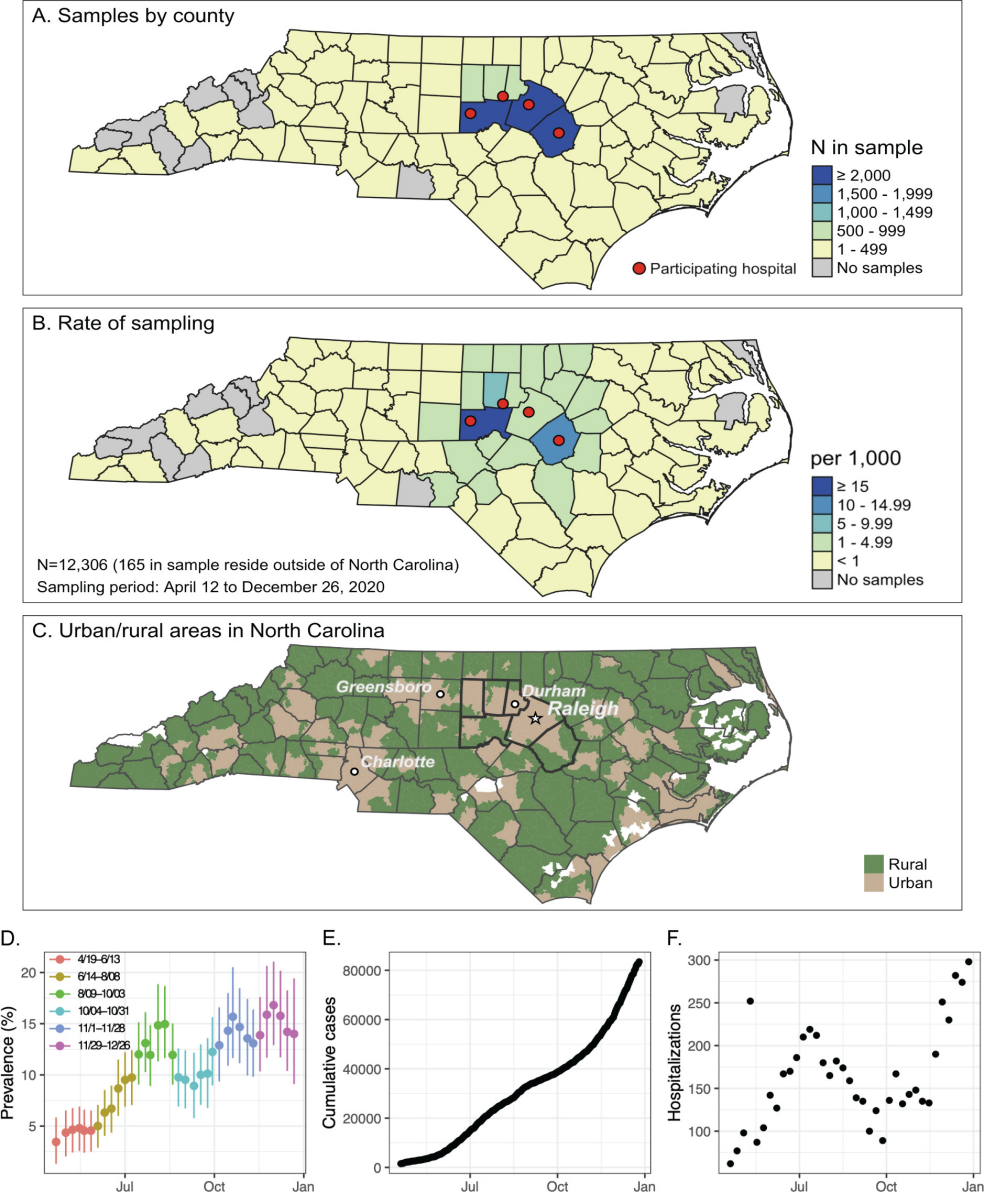
Catchment area for sample collection and trends in prevalence and cases over the study time period. Remnant samples were collected from hospital clinical laboratories from each of the four sites indicated by the red dots. (A) Number of samples collected by count as well as (B) the rate of sampling. (C and D) North Carolina urban and rural areas displayed for comparison in map (C) as defined by U.S. Census zip code tabulation areas (42). (D) Weekly posterior mean seroprevalence estimates and 95% credible intervals for the study period of 19 April to 26 December of the hospital samples by ELISA plotted over time over the course of the study period. (E) Cumulative daily COVID-19 PCR+ cases from the six-county area from 19 April to 26 December and (F) weekly COVID-19 hospitalizations in the six-county area from 19 April to 26 December from the NC Department of Health and Human Services.

From 19 April 2020 to 26 December 2020, after excluding duplicate samples, 12,471 remnant samples were obtained from four University of North Carolina (UNC) Health hospitals in central NC. The six counties most heavily sampled were Orange, Johnson, Chatham, Wake, Durham, and Alamance, with 9,013 (72.2%) of individuals residing in these counties (Fig. 1). Furthermore, out of 12,471 individuals in the study, 3,764 reside in rural ZIP codes (30.2%), 8,701 reside in urban ZIP codes (69.8%), and 6 individuals were unable to be determined because the ZIP code in the information was a 4-digit code. The study consists of 7,070 females (56.7%) and 5,400 males (43.3%), which is similar to the demographics of this region (Table S3). Samples from the youngest age group (5 to 17 years) were underrepresented, comprising only 6% of the cohort, even though this age group represents over 18% of the study area’s population. Over 90% of study individuals were insured, with 7.5% falling into the self-pay category. By comparison, 10.4% of the total state population is uninsured (24). The majority of sampled individuals (5,560, 44.6%) were seen at UNC Memorial Hospital, 2.6% were acute or trauma cases, and 4.1% had a visit diagnosis of fever or respiratory symptoms (Table S4). Overall, approximately 1% of patients had an associated COVID-19 visit diagnosis, with a significant difference between inpatients (3.14%) and outpatients (0.24%) (Chi-squared test; *P* < 0.0001) (Table S5).

10.1128/mSphere.00841-21.6 TABLE S3 Study participants by demographic factors of interest*^a^*.^*a*^Because of how the NC census reports data, the sex and age breakdowns of the 6-county demographics include only individuals over the age of 4 (including those over the age 99), but the race/ethnicity breakdown includes individuals of all ages. Additionally, the 65 to 99 age category is actually age 65+ for the 6-county demographics. Download Table S3, DOCX file, 0.01 MB. Copyright © 2022 Lopez et al. 2022 Lopez et al. https://creativecommons.org/licenses/by/4.0/ This content is distributed under the terms of the Creative Commons Attribution 4.0 International license.

10.1128/mSphere.00841-21.7 TABLE S4 Study individual numbers by clinical factors. Download Table S4, DOCX file, 0.01 MB. Copyright © 2022 Lopez et al. 2022 Lopez et al. https://creativecommons.org/licenses/by/4.0/ This content is distributed under the terms of the Creative Commons Attribution 4.0 International license.

10.1128/mSphere.00841-21.8 TABLE S5 Rates of COVID-19 visit codes for inpatients and outpatients. Download Table S5, DOCX file, 0.01 MB. Copyright © 2022 Lopez et al. 2022 Lopez et al. https://creativecommons.org/licenses/by/4.0/ This content is distributed under the terms of the Creative Commons Attribution 4.0 International license.

### Seroprevalence estimates.

In order to understand how seroprevalence in central NC changed over time rather than obtain an estimate averaged across the year, we arbitrarily divided the 9-month period of the study into four time periods, each approximately 9 weeks in duration. We utilized an receptor binding domain (RBD) Ig enzyme-linked immunosorbent assay (ELISA) with sensitivity of 89.7% (95% confidence interval [CI], 84.7 to 94.6) and specificity 99.3 (95% CI, 98.3 to 100) to estimate seroprevalence in the population (Table S2). The Bayesian hierarchical model (BHM)-derived seroprevalence estimates increased from almost 3% in April to June to almost 13% in November/December (Table 1). Seroprevalence estimates peaked in early August following a hospitalization peak in mid-July (Fig. S3A and C). Cumulative PCR-positive COVID-19 cases reported by the state for these six counties increased over the study period (Fig. S3B), with the most rapid accumulation of cases occurring from November through December. Sample positivity surged again in the final 9-week period. These peaks and declines were not affected when samples with International Classification of Diseases 10th revision (ICD-10) visit codes for “COVID-19” or those we identify as “respiratory disease” are removed from the data set (data not shown).

**TABLE 1:** Cohort prevalence estimates^*a*^

Trait	Percent positivity by period (mo/day) (%)				Bayesian hierarchical model prevalence estimates by period (mo/day) (%)							
	4/19–6/20	6/21–8/22	8/23–10/24	10/25–12/26	4/19–6/20		6/21–8/22		8/23–10/24		10/25–12/26	
					Estimate	95% CI	Estimate	95% CI	Estimate	95% CI	Estimate	95% CI
Overall	5.4	11.8	10.9	13.9	2.9	1.8–4.5	10.2	8.5–12.2	9.1	7.2–11.2	12.8	10.6–15.2
Age (yrs)												
5–17	4.2	9.7	8.4	12.1	1.9	0.5–4.3	8.0	3.9–13.2	6.8	2.8–11.9	11.4	6.3–17.4
18–49	5.9	13.4	10.9	15.5	3.5	2.1–5.3	12.1	9.5–14.8	9.1	6.3–12.0	14.5	11.1–18.0
50–64	5.9	12.4	12.5	12.7	3.8	2.1–5.8	10.9	8.4–13.7	11.1	8.2–14.3	11.3	8.0–14.8
65–99	4.6	10.2	10.1	13.7	1.7	0.3–3.7	8.4	6.3–10.8	8.1	5.5–10.9	12.7	9.5–16.2
Sex												
Female	4.6	11.9	10.1	14.9	2.2	1.1–3.7	10.4	8.3–12.6	8.1	5.8–10.5	13.9	11.2–16.7
Male	6.4	11.5	11.9	12.5	3.9	2.3–5.9	10.1	7.9–12.3	10.5	8.0–13.1	11.3	8.5–14.3
Race/ethnicity^*b*^												
NL White	3.8	8.2	8.8	10.5	1.5	0.5–2.8	6.2	4.4–8.1	6.7	4.6–8.9	9.0	6.7–11.5
NL Black	5.6	13.9	12.9	17.1	2.5	0.6–4.9	12.7	9.7–15.8	11.6	8.3–15.1	16.3	12.2–20.8
NL Other	5.7	12.3	12.0	12.9	2.1	0.1–6.0	10.8	6.2–16.4	9.9	5.1–15.4	11.4	6.3–17.3
Latinx	16.6	34.0	21.2	33.5	15.3	10.9–20.2	35.7	29.2–42.7	20.7	14.4–27.8	35.0	26.7–43.8
In-/outpatient												
Outpatient	4.5	10.1	8.3	12.0	2.1	1.1–3.5	8.3	6.4–10.2	6.1	4.1–8.3	10.7	8.4–13.0
Inpatient	7.6	16.0	16.0	19.1	4.9	2.9–7.3	15.2	12.3–18.2	15.2	12.0–18.6	18.5	14.6–22.8
Payor												
Private	5.2	9.8	9.3	12.1	2.9	1.5–4.6	8.2	6.1–10.6	7.6	5.2–10.2	10.9	8.1–13.8
Public	5.2	11.1	10.9	14.0	2.5	1.2–4.3	9.4	7.4–11.6	9.1	6.9–11.6	12.9	10.2–15.8
Self-pay	4.5	20.9	16.3	19.3	2.0	0.4–4.4	20.7	15.1–26.7	15.0	8.7–22.2	19.2	12.2–27.2
Other/unknown	21.7	38.2	36.4	46.2	20.8	11.6–31.2	38.9	26.8–52.2	34.9	16.8–55.7	46.6	27.9–66.5

a Raw seropositivity (%) and posterior mean seroprevalence estimates (%) from our Bayesian hierarchical model with 95% credible intervals (lower bound, upper bound).

b NL, non-Latinx.

10.1128/mSphere.00841-21.5 TABLE S2 ELISA validation data*^a^*.^*a*^CI, confidence interval; CP, convalescent plasma; TB, tuberculosis. Download Table S2, DOCX file, 0.01 MB. Copyright © 2022 Lopez et al. 2022 Lopez et al. https://creativecommons.org/licenses/by/4.0/ This content is distributed under the terms of the Creative Commons Attribution 4.0 International license.

### Prediction for seropositivity by demographic and clinical characteristics.

Latinx-identifying individuals have higher SARS-CoV-2 seroprevalence, increasing from 15 to 35% compared to non-Latinx white individuals, which have only ~2 to 9% seroprevalence over the study period (Table 1). Individuals with “other/unknown” or “self-pay” insurance status had a higher estimated seroprevalence (increasing from ~21 to 47% seropositive or ~2 to 19% seropositive, respectively) than those with private or public health insurance (~3 to 13% seropositive). 30% of Latinx individuals in this study were in the other/unknown or self-pay health insurance categories. Within these likely underinsured categories, 27% are Latinx despite only accounting for ~8% of our study population (Table S6). We found little seroprevalence variation by age group. For example, 5-to 17-year-olds begin at 4.2% SARS-CoV-2 seropositive in April 2020, increasing to 12.1% seropositive by December 2020. This compares to the highest-seroprevalence age group, 18- to 49-year-olds, starting at 5.9% in April and increasing to 15.5% in December 2020. Similarly, female and male seroprevalence begin at 4.6% and 6.4% seropositive in April 2020, increasing to 14.9% and 12.5%, respectively.

10.1128/mSphere.00841-21.9 TABLE S6 Count and percentage in each insurance category by race/ethnicity. Download Table S6, DOCX file, 0.01 MB. Copyright © 2022 Lopez et al. 2022 Lopez et al. https://creativecommons.org/licenses/by/4.0/ This content is distributed under the terms of the Creative Commons Attribution 4.0 International license.

We calculated conditional odds ratios for each clinical and demographic variable we collected, thus measuring the effect of that variable alone while holding other variables constant (Table 2). Calculation of the conditional odds ratio allows us to compare the odds of seropositivity for any one demographic to the odds of seropositivity for another demographic (e.g., the odds of seroprevalence in males compared to females of the same insurance status, age, ethnoracial category, and whether they are inpatient or outpatient). Latinx individuals had the highest odds of SARS-CoV-2 exposure throughout the study period compared to non-Latinx white individuals, with the odds ratio (OR) of 6.56 overall (4.66 to 9.44) ranging from 15.25 (7.01 to 39.28) in the first 9-week period to 5.22 (3.20 to 8.70) in the last 9-week period of the study. Individuals with unknown insurance status also had an elevated odds ratio of seropositivity at 3.60 (2.21 to 5.89) compared to those with private insurance status. Over the entire period of the study, other groups with increased odds ratios include non-Latinx black individuals at 1.81 (1.33 to 2.41) and inpatients at 2.16 (1.73 to 2.77).

**TABLE 2:** Conditional odds ratios of being SARS-CoV-2 seropositive over the study period^*a*^

Trait	Apr 19–Jun20		Jun 21–Aug 22		Aug 23–Oct 24		Oct 25–Dec 26		Apr 19–Dec 26 (overall)	
	OR	95% CI	OR	95% CI	OR	95% CI	OR	95% CI	OR	95% CI
Sex										
Female										
Male	**1.92**	**1.05–3.86**	0.93	0.70–1.22	1.15	0.81–1.65	0.71	(0.50, 1.00)	1.1	(0.89, 1.38)
Race/ethnicity^*b*^										
NL white										
NL black	1.59	0.52–3.91	**2.11**	**1.50–3.05**	**1.72**	**1.13–2.67**	**1.85**	**1.25–2.75**	**1.81**	**1.33–2.41**
NL other	1.28	0.13–5.73	**1.95**	**1.05–3.44**	1.86	0.90–3.61	1.38	0.73–2.46	1.59	0.85–2.56
Latinx	**15.25**	**7.01–39.28**	**7.29**	**4.77–11.33**	**3.2**	**1.83–5.65**	**5.22**	**3.20–8.70**	**6.56**	**4.66–9.44**
Age (yrs)										
5–17										
18–49	2.11	0.74–7.20	1.38	0.70–2.93	1.06	0.49–2.59	1.26	0.67–2.50	1.4	0.93–2.17
50–64	2.98	1.00–10.55	1.64	0.81–3.54	1.45	0.68–3.51	1.05	0.54–2.13	**1.65**	**1.08–2.62**
65–99	1.66	0.37–6.16	1.64	0.80–3.60	1.23	0.57–3.05	1.46	0.75–2.98	1.49	0.91–2.38
In/out patient										
Outpatient										
Inpatient	**2.32**	**1.28–4.39**	**1.87**	**1.40–2.54**	**2.61**	**1.78–4.04**	**1.94**	**1.36–2.78**	**2.16**	**1.73–2.77**
Payor										
Private										
Public	0.83	0.41–1.61	0.95	0.66–1.38	1	0.63–1.61	0.89	0.57–1.37	0.92	0.71–1.17
Self-pay	0.31	0.08–0.89	**1.84**	**1.14, 2.91**	1.66	0.84–3.15	1.23	0.66–2.23	1.04	0.67–1.50
Other/unknown	**3.08**	**1.17–8.11**	**3.57**	**1.71–7.31**	**3.59**	**1.20–10.35**	**4.25**	**1.52–12.02**	**3.6**	**2.21–5.89**

a Data are broken down into three 9-week-long periods in central North Carolina. Odds ratios of seropositivity calculated from the BHM with 95% credible intervals (lower bound, upper bound) are reported where the baseline groups for comparison are female, non-Latinx white, age 5 to 17, outpatient, and private insurance. Odds ratios that do not overlap a value of 1 are bolded.

b NL, non-Latinx.

### SARS-CoV-2 RBD-positive subset analysis.

**FIG 2:**
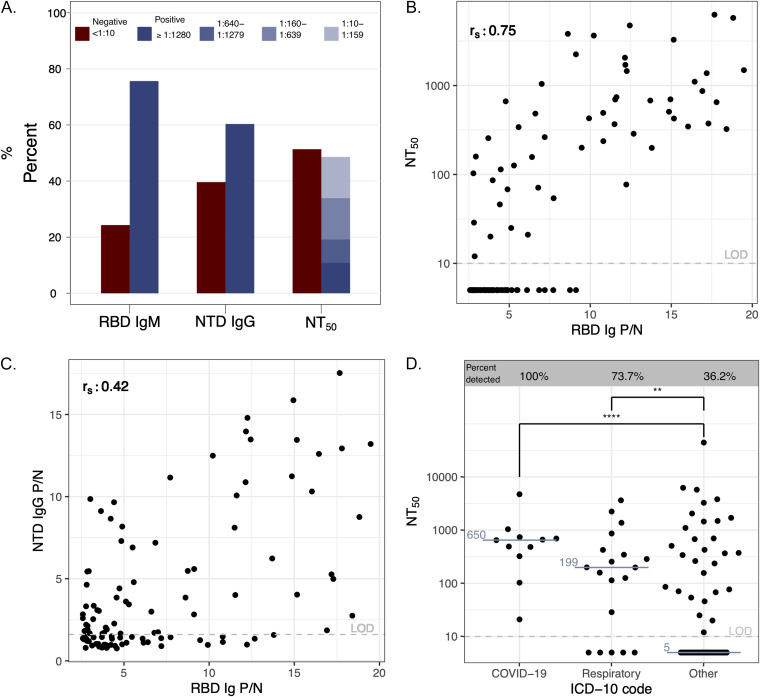
Antibody repertoires in an RBD Ig-positive subset; 110 RBD Ig-positive samples were chosen at random to undergo SARS-2 antibody repertoire analysis. (A) Percentage of individuals with RBD IgM, NTD IgG, and functionally neutralizing antibodies (NT50). (B) Correlation plot of NT_50_ and RBD Ig. (C) Correlation plot of NTD IgG and RBD Ig; r_s_ = Spearman correlation coefficient displayed in the top left of panels B and C. (D) NT_50_ values for each diagnosis binning category based on ICD-10 codes. Medians are shown in blue. Two-tailed Mann-Whitney test; ****, *P* < 0.0001; **, *P* = 0.0078.

To determine the SARS-CoV-2 antibody repertoire in RBD total Ig seropositive individuals, we randomly selected 110 of the above-described positive samples in this cohort for further analysis. About 75% of individuals were positive for RBD IgM, 60% had N-terminal domain (NTD) IgG antibodies, and about 50% had detectable neutralizing antibodies (Fig. 1A). Of the participants with detectable functionally neutralizing antibodies, 23% had a high titer of >1:1,280, 47% had a moderate titer of 1:160 to 1:1,279, and 30% had a lower titer of 1:10 to 1:159. Furthermore, RBD Ig positive to negative (P/N) antibody signal correlated more strongly with functionally neutralizing antibody levels (Fig. 1B) than NTD IgG signal (Fig. 1C). We also found that 36% (29/80) of those in this subset with an ICD-10 code binned as “other” had detectable neutralizing antibodies, while 83% (25/30) of individuals with an ICD-10 code of “COVID-19” or what we identify as “respiratory disease” had neutralizing antibodies (Fig. 1D). There was substantial agreement between the RBD Ig ELISA results reported here and 150 study individuals for which a clinical SARS-CoV-2 nucleocapsid IgG (Abbott assay) was available (Cohen’s kappa = 0.700) (Fig. 2; Table S6).

## DISCUSSION

Using clinical remnant samples from more than 12,000 unique health care-seeking individuals in central NC between April and December 2020, we estimate a significant increase in overall seroprevalence from 2.9% (95% CI, 1.8 to 4.5) at the start of the study period, to 12.8% (95% CI, 10.6 to 15.2) at the end of the study period, approximately 9 months after the first case in the state. Our data support a national study, also using remnant inpatient and outpatient clinical samples that estimated a state-wide seroprevalence of 6.8% (4.8 to 8.9) from samples collected in NC in mid-September 2020 (7), which is close to our estimate of 9.1% (7.1 to 11.2) from samples collected from 23 August through 24 October 2020 in central NC. Interestingly, overall seroprevalence slightly declined in the summer, which is likely attributable to a surge of positivity in Johnston County. In Johnston County, the estimated seroprevalence more than doubled between the first and second 9-week periods, which then fell to 12.7% in the third period (Table S1). In contrast, seroprevalence estimates from the other three locations steadily increased during the first three periods. The end-of-study prevalence identified here is substantially higher than the cumulative number of confirmed cases identified by PCR testing in the same county region at the same date, though determining the degree to which the identified cases undercount true infections requires more representative sampling. Our findings demonstrate both the feasibility and potential for broad application of using routinely collected blood samples to estimate community prevalence during a new outbreak. Now that a significant number of individuals have been vaccinated, it will be necessary to assay in parallel for antibodies to proteins in addition to spike (25).

10.1128/mSphere.00841-21.4 TABLE S1 Bed numbers and raw antibody test positivity (percent) for each participating hospital (number of beds and county location of each hospital in this study.) Download Table S1, DOCX file, 0.01 MB. Copyright © 2022 Lopez et al. 2022 Lopez et al. https://creativecommons.org/licenses/by/4.0/ This content is distributed under the terms of the Creative Commons Attribution 4.0 International license.

The conditional odds ratios we calculated assume that all other variables are held constant while estimating the effect of one demographic variable at a time. We found that Latinx individuals had the highest odds of SARS-CoV-2 seropositivity, followed by non-Latinx black individuals, corroborating observations within the United States, including urban centers as well as state-wide studies within and outside the American South (4, 8–10, 13). In nearby Virginia among outpatients as of August 2020, Hispanics were found to have about 6-fold increased seropositivity compared to white individuals (10). Similarly, in Houston, Texas, by September 2020 as measured by representative population sampling, rates of seropositivity were about 3-fold higher among both non-Hispanic black and Hispanic individuals compared to non-Hispanic white individuals (8). Around that same time period, in our study, we found a 3- to 4-fold higher seropositivity among Latinx individuals compared to non-Latinx white individuals, which supports the previous findings in Texas and Virginia. We observe that high odds ratios by race and ethnicity decrease over time, consistent with a paradigm of spread first among individuals with high exposure risk and subsequently to lower-risk individuals. Residential segregation, crowded households, socioeconomic disadvantage, mass incarceration, and inequities in access to insurance, health care, and access to testing, vaccination, and treatments have all been cited as factors that have contributed to the large and sustained racial and ethnic disparities in COVID-19 in the United States (18, 20, 26–28). We also observed that individuals that self-pay for their health care or otherwise had unknown insurance status have higher SARS-CoV-2 seropositivity and odds ratios. The significant overlap in the Latinx population and these insurance categories is concerning because the high odds ratios and seroprevalence in these categories can lead to much higher exposure risk among the significant number of underinsured Latinx individuals (29). Finally, given our predominantly urban population and the inclusion of the second-largest city in North Carolina in our main catchment area, the ethnoracial disparities we identify may be associated with urban living centers; however, given a recent study that finds rural versus urban U.S. populations to be not as ethnoracially distinct as previously thought, more research into this area looking at living conditions, occupation, poverty, etc. is needed to understand if there is such an association (30).

Some studies of PCR-positive symptomatic COVID-19 cases have reported neutralizing antibody responses in these individuals (31), yet we observed that 51% of individuals in our RBD-positive subset analysis did not have detectable neutralizing antibodies, corroborating the low rates of neutralizing antibodies observed in a prevalence study done by Aziz et al. (32). When differentiating neutralizing antibody titers by ICD-10 code, the majority of all individuals with a “respiratory disease” or “COVID-19” diagnosis had developed neutralizing antibodies, compared to fewer than 40% without these diagnoses. Individuals with mild or asymptomatic disease are likely to not have a diagnosis of COVID-19 or respiratory symptoms. Limited duration of infection or lack of hematogenous dissemination in mild or asymptomatic disease may explain why these individuals have undetectable neutralization titers (11, 33). We also found that 75% of this subset had RBD IgM antibodies, indicating that their infections likely occurred within 3 months of blood draw (33). Furthermore, a majority of individuals in this subset had detectable NTD IgG antibodies; the NTD has recently been found to be an important target for the B.1.1.7, B.1.351, B.1.1.28.1, and B.1.617 SARS-CoV-2 variants (34, 35). Neutralizing antibodies have been identified as a correlate of protection against SARS-CoV-2 following vaccination (36, 37). If clinically unapparent SARS-CoV-2 infection inconsistently leads to a neutralizing antibody response, vaccination even among individuals with serologic evidence of infection should continue to be recommended.

The primary limitation of this study is that the study population composed of individuals accessing care at UNC area hospitals and clinics when many nonemergent procedures were postponed likely differs from the overall population in central NC in ways that are not captured in demographic data (e.g., overall health status). Accordingly, we have chosen to not weight our data set to demographics of the counties in the main catchment area and therefore do not provide community estimates of seroprevalence. A more representative sampling methodology is needed (38). Declining antibody over this time period to undetectable levels is unlikely, although little is known about antibody levels over time in the asymptomatic population (31). We do not further differentiate odds ratios by combinations of race and ethnicity because the small number of individuals per subdivision prevented statistical interpretation. Finally, we do not know who is truly uninsured because lack of insurance is not a recordable category within the electronic medical records (EMR). Although SARS-CoV-2 seroprevalence of health care-seeking individuals is an imperfect comparison to the general population, we maintain that it is a useful sentinel population to understand overall trends, especially when studying rural regions lacking well-funded community health initiatives.

Based on our seroprevalence estimates in this study population, cumulative case numbers confirmed by molecular diagnostics are underrepresenting the true number of infections. Vaccination and public health measures such as physical distancing and mask-wearing where appropriate should continue to be prioritized in order to lower the transmission of SARS-CoV-2 and subsequent loss of life. Our findings of significantly higher odds of SARS CoV-2 seropositivity among Latinx populations corroborate numerous studies describing large racial and ethnic disparities in SARS-CoV-2 infection, morbidity, and mortality in the United States (4, 10, 13). Finally, vaccination programs should address structural and occupational factors that drive racial and ethnic disparities in health outcomes in the United States to ensure that individuals at particularly high exposure risk of SARS-CoV-2 have timely access to SARS-CoV-2 vaccination regardless of prior infection.

## MATERIALS AND METHODS

### Sampling strategy and data collection.

Remnant plasma and serum samples were collected from four hospital-based clinical laboratories affiliated with the University of North Carolina (UNC) Health system (Table S1). These laboratories receive and process clinical samples from inpatient units as well as outpatient clinics in central NC. Each week, laboratory staff at each location, who were unaware of the patient’s clinical status or hospitalization/visit details, selected up to 300 remnant samples belonging to individuals 5 to 99 years of age. Samples were collected between 19 April 2020 to 26 December 2020. Medical record numbers were recorded for each sample, and study duplicates were discarded. We abstracted the following demographic and clinical data from electronic medical records (EMR, Epic)—age, legal sex, ethnicity, race, address, including city, state, and ZIP code, insurance coverage, insurance type, inpatient or outpatient status, encounter diagnosis by International Classification of Diseases 10th revision (ICD-10 code), inpatient date of discharge, and whether or not COVID-19 testing was performed within a 30-day window prior to study sample collection. The study catchment area includes the county of the first confirmed case in NC (39), which was reported on 3 March 2020. By 26 December 2020, there were 83,457 cumulative total PCR-confirmed SARS-CoV-2 cases in the main study catchment area (4.3% of the population), with 1,133 confirmed COVID-19 related deaths (40, 41). All data for this study were collected under UNC Institutional Review Board (IRB) no. 20-0791, which is conducted under good clinical research practices (GCP) and compliant with IRB oversight. The requirement for written informed consent was waived due to the use of routinely collected samples. Deidentified samples used for assay validation were collected under UNC IRB no. 20-1141, 20-0913, and 08-0895.

Urban/rural classification of a ZIP code was determined by following Census Bureau guidance for classification of ZIP code tabulation areas (ZCTA), a census enumeration unit based on aggregating census blocks to ZIP code areas (42). Briefly, the Census Bureau reports estimates of the population in each ZCTA that is either urban or rural. If 50% or more of the population in a given ZCTA is urban, the ZCTA was classified as urban. All ZCTAs were classified as either rural or urban using this method. If an individual in the study lives in an urban ZIP code, they were classified as urban.

### Enzyme-linked immunosorbent assays.

A total Ig ELISA (combined IgG, IgM, and IgA) and IgM SARS-CoV-2 RBD ELISA, neither of which cross-react with common endemic human coronaviruses, were used in this study as previously described (43). Briefly, the SARS-CoV-2 RBD (amino acids 331 to 528, GenBank accession no. QIS60558) containing three purification tags (6× histidine, Halo, and TwinStrep) was cloned into the pαH mammalian expression vector and expressed in Expi293 cells (Thermo Fisher) and then purified using nickel-nitrilotriacetic acid agarose. The spike protein N-terminal domain (NTD) antigen used in ELISAs for the positive sample subset analysis (amino acids 16 to 305, GenBank accession no. P0DTC2) was cloned similarly. All ELISA measurement was conducted in duplicate, and duplicate values with variance of >25% and/or one value above-described assay cutoff were repeated. A correlation plot shows the use of 140 COVID-19 PCR-confirmed cases between our RBD Ig positive to negative (P/N) ratios and the neutralization assay described below (Fig. S1). We chose to use a SARS-CoV-2 spike-based assay to estimate prevalence due to the growing concern about the use and performance of nucleocapsid IgG assays in individuals with asymptomatic or mild disease, as these assays appear to be less sensitive in asymptomatic disease cohorts but have comparable sensitivity to spike or RBD assays in symptomatic disease cohorts (44–47).

10.1128/mSphere.00841-21.2 FIG S1 Correlation plot between RBD Ig P/N and neutralization assay. The Spearman correlation coefficient was obtained (r_s_); *P* < 0.0001. Download FIG S1, TIF file, 0.08 MB. Copyright © 2022 Lopez et al. 2022 Lopez et al. https://creativecommons.org/licenses/by/4.0/ This content is distributed under the terms of the Creative Commons Attribution 4.0 International license.

### Nucleocapsid protein ELISA.

Detection of IgG antibody to SARS-CoV-2 N antigen was performed with the emergency use authorization (EUA)-approved SARS-CoV-2 IgG assay (Abbott Laboratories) on the Abbott Architect i2000SR immunoassay analyzer as previously described (33). This assay has a sensitivity of 69.0% 8 days or more post-symptom onset and an overall specificity of 99.6% (48).

### SARS-CoV-2 neutralization assays.

To further characterize the SARS-CoV-2 antibody responses of this study, viral neutralization assays were obtained for 110 ELISA-positive samples collected between 21 April 2020 and 8 July 2020 that were selected randomly using the sample_n() function of the dplyr R package. Luciferase-expressing, full-length SARS-CoV-2 isolate WA1 strain (GenBank accession no. MT020880) was engineered and recovered via reverse genetics and used to determine the titer of serially diluted sera on Vero E6 USAMRID cells as described previously (49). The sample dilution at which a 50% reduction in relative light units (RLU) was observed relative to that of the virus control wells was used as the 50% neutralization titer (NT_50_) for that sample.

### Statistical methods and analyses.

For the RBD-based assay, we calculated P/N ratios for each sample, defined as the average optical density (OD) of the sample divided by the average OD of the negative control in its respective ELISA plate. Following the CDC recommendation to set specificity to 99.5%, we chose the 0.995 quantile of the P/N ratio for the negative validation samples as the P/N cutoff (50). Validation sera were collected from 145 PCR-confirmed SARS-CoV-2-positive cases from the laboratories of Jennifer Dan, Alessandro Sette, and Shane Crotty at La Jolla Institute of Immunology and 274 negative controls collected prior to 2020 (Table S2).

We fit two statistical models to estimate seroprevalence. First, we fit a Bayesian (51) autoregressive logistic model to estimate weekly prevalence across the 9-month study period while accounting for uncertainty in the assay specificity and sensitivity calculated by the internal laboratory validation. Second, we fit a Bayesian logistic regression model to estimate the prevalence and conditional odds ratios by subpopulation with main effects for sex, race/ethnicity, age, in-/outpatient status, and health insurance payor, while again accounting for uncertainty in the assay test characteristics (Table S2). Each group was compared to females, non-Latinx white, ages 5 to 17, outpatient, and private payor health insurance status as respective baseline categories because these had both high sample numbers and lowest seroprevalence within each category. Details are given in supplemental Text S1–Bayesian seroprevalence models with unknown sensitivity and specificity. These Bayesian hierarchical models (BHM) simultaneously model study data and validation data to produce prevalence estimates and credible intervals that reflect both uncertainty due to the finite study sample as well as the uncertainty in the sensitivity and specificity of the ELISA, with statistical uncertainty represented by 95% credible intervals.

10.1128/mSphere.00841-21.1 TEXT S1 This section includes our methodology for the hierarchical Bayesian modeling and logistic regression modeling, quantifying uncertainty as well as demographic data categorization. This section also holds the bibliography for all of our supplemental information. Download Text S1, DOCX file, 0.04 MB. Copyright © 2022 Lopez et al. 2022 Lopez et al. https://creativecommons.org/licenses/by/4.0/ This content is distributed under the terms of the Creative Commons Attribution 4.0 International license.

10.1128/mSphere.00841-21.10 TABLE S7 Subset of individuals with recorded Abbott IgG*^a^*.^*a*^RBD Ig ELISA results for patients who received a UNC hospital lab-based Abbott nucleocapsid IgG ELISA within 1 month prior to study enrollment. Download Table S7, DOCX file, 0.01 MB. Copyright © 2022 Lopez et al. 2022 Lopez et al. https://creativecommons.org/licenses/by/4.0/ This content is distributed under the terms of the Creative Commons Attribution 4.0 International license.
