# Supplementary material for: Ethnoracial Disparities in SARS-CoV-2 Seroprevalence in a Large Cohort of Individuals in Central North Carolina from April to December 2020
Source: mSphere. 2022 May 19;7(3):e00841-21. doi: 10.1128/msphere.00841-21 (PMC9241523; doi:10.1128/msphere.00841-21)
Supplement: TABLE S3 [file msphere.00841-21-s0004.docx]

| **Table S3. Study participants by demographic factors of interest.** | | | | | | | | |  |
| --- | --- | --- | --- | --- | --- | --- | --- | --- | --- |
|  | 4/19-6/20 | | 6/21-8/22 | | 8/23-10/24 | | 10/25-12/26 | | 6-county  Demographics (%) |
|  | N | (%) | N | (%) | N | (%) | N | (%) |  |
| **Sex** | | | | | | | | |  |
| Female | 2227 | 56.2 | 2175 | 56.5 | 1392 | 55.5 | 1276 | 59.4 | 51.8 |
| Male | 1735 | 43.8 | 1676 | 43.5 | 1118 | 44.5 | 871 | 40.6 | 48.2 |
| Unreported | 1 | 0.0 | 0 | 0.0 | 0 | 0.0 | 0 | 0.0 | — |
| **Age** | | | | | | | | |  |
| 5-17 | 289 | 7.3 | 176 | 4.6 | 155 | 6.2 | 166 | 7.7 | 18.4 |
| 18-49 | 1419 | 35.8 | 1190 | 30.9 | 817 | 32.5 | 756 | 35.2 | 48.7 |
| 50-64 | 1080 | 27.3 | 1082 | 28.1 | 682 | 27.2 | 568 | 26.5 | 19.7 |
| 65-99 | 1175 | 29.6 | 1403 | 36.4 | 856 | 34.1 | 657 | 30.6 | 13.1 |
| **Race/Ethnicity** | | | | | | | | |  |
| NL White | 2443 | 61.6 | 2452 | 63.7 | 1532 | 61.0 | 1308 | 60.9 | 59.7 |
| NL Black | 954 | 24.1 | 875 | 22.7 | 590 | 23.5 | 474 | 22.1 | 21.0 |
| NL Other | 229 | 5.8 | 227 | 5.9 | 209 | 8.3 | 201 | 9.4 | 8.2 |
| Latinx | 337 | 8.5 | 297 | 7.7 | 179 | 7.1 | 164 | 7.6 | 11.1 |
| **In/Out patient** | | | | | | | | |  |
| Inpatient | 1179 | 29.8 | 1098 | 28.5 | 835 | 33.3 | 593 | 27.6 | — |
| Outpatient | 2772 | 69.9 | 2747 | 71.3 | 1674 | 66.7 | 1553 | 72.3 | — |
| Unknown | 12 | 0.3 | 6 | 0.2 | 1 | 0.0 | 1 | 0.0 | — |
| **Payor** | | | | | | | | |  |
| Public | 2147 | 54.2 | 2229 | 57.9 | 1435 | 57.2 | 1079 | 50.3 | — |
| Private | 1371 | 34.6 | 1299 | 33.7 | 900 | 35.9 | 907 | 42.2 | — |
| Self-Pay | 376 | 9.5 | 268 | 7.0 | 153 | 6.1 | 135 | 6.3 | — |
| Other/Unknown | 69 | 1.7 | 55 | 1.4 | 22 | 0.9 | 26 | 1.2 | — |
