# Supplementary material for: Ethnoracial Disparities in SARS-CoV-2 Seroprevalence in a Large Cohort of Individuals in Central North Carolina from April to December 2020
Source: mSphere. 2022 May 19;7(3):e00841-21. doi: 10.1128/msphere.00841-21 (PMC9241523; doi:10.1128/msphere.00841-21)
Supplement: TABLE S4 [file msphere.00841-21-s0005.docx]

| **Table S4.** **Study Individual Numbers by Clinical Factors.** | | | | | | | | |
| --- | --- | --- | --- | --- | --- | --- | --- | --- |
|  | **4/19-6/20** | | **6/21-8/22** | | **8/23-10/24** | | **10/25-12/26** | |
|  | N | (%) | N | (%) | N | (%) | N | (%) |
| **Hospital** | | | | | | | | |
| Chatham Hospital | 788 | 19.9 | 903 | 23.4 | 182 | 7.3 | 195 | 9.1 |
| UNC Hospitals | 1645 | 41.5 | 1717 | 44.6 | 1262 | 50.3 | 936 | 43.6 |
| Johnston Hospital | 704 | 17.8 | 730 | 19.0 | 402 | 16.0 | 247 | 11.5 |
| Rex Hospital | 826 | 20.8 | 501 | 13.0 | 664 | 26.5 | 769 | 35.8 |
| **In/Outpatient** | | | | | | | | |
| Inpatient | 1179 | 29.8 | 1098 | 28.5 | 835 | 33.3 | 593 | 27.6 |
| Outpatient | 2772 | 69.9 | 2747 | 71.3 | 1674 | 66.7 | 1553 | 72.3 |
| Unknown | 12 | 0.3 | 6 | 0.2 | 1 | 0.0 | 1 | 0.0 |
| **Visit type** | | | | | | | | |
| Traumatic | 122 | 3.1 | 107 | 2.8 | 59 | 2.4 | 41 | 1.9 |
| Not traumatic | 3385 | 85.4 | 3370 | 87.5 | 2022 | 80.6 | 1557 | 72.5 |
| Unknown | 456 | 11.5 | 374 | 9.7 | 429 | 17.1 | 549 | 25.6 |
| **Condition** | | | | | | | | |
| Respiratory | 196 | 4.9 | 167 | 4.3 | 94 | 3.7 | 57 | 2.7 |
| COVID-19 | 36 | 0.9 | 41 | 1.1 | 28 | 1.1 | 27 | 1.3 |
| Other | 3275 | 82.6 | 3269 | 84.9 | 1959 | 78.0 | 1514 | 70.5 |
| Unknown | 456 | 11.5 | 374 | 9.7 | 429 | 17.1 | 549 | 25.6 |
| **Payor** | | | | | | | | |
| Public | 2147 | 54.2 | 2229 | 57.9 | 1435 | 57.2 | 1079 | 50.3 |
| Private | 1371 | 34.6 | 1299 | 33.7 | 900 | 35.9 | 907 | 42.2 |
| Self-Pay | 376 | 9.5 | 268 | 7.0 | 153 | 6.1 | 135 | 6.3 |
| Other/Unknown | 69 | 1.7 | 55 | 1.4 | 22 | 0.9 | 26 | 1.2 |
