# Supplementary material for: Ethnoracial Disparities in SARS-CoV-2 Seroprevalence in a Large Cohort of Individuals in Central North Carolina from April to December 2020
Source: mSphere. 2022 May 19;7(3):e00841-21. doi: 10.1128/msphere.00841-21 (PMC9241523; doi:10.1128/msphere.00841-21)
Supplement: TABLE S5 [file msphere.00841-21-s0006.docx]

| **Table S5. Rates of COVID-19 Visit Codes for Inpatients and Outpatients.** | | |
| --- | --- | --- |
|  | **Inpatient** | **Outpatient** |
| **COVID-19 Visit Code** | 115/3667 (3.14%) | 17/6994 (0.24%) |
