# Supplementary material for: Ethnoracial Disparities in SARS-CoV-2 Seroprevalence in a Large Cohort of Individuals in Central North Carolina from April to December 2020
Source: mSphere. 2022 May 19;7(3):e00841-21. doi: 10.1128/msphere.00841-21 (PMC9241523; doi:10.1128/msphere.00841-21)
Supplement: TABLE S2 [file msphere.00841-21-s0003.docx]

| **Table S2. ELISA Validation Data** | | |
| --- | --- | --- |
|  | **% Sensitivity (95% CI)** | **% Specificity (95% CI)** |
| RBD total Ig ELISA  (≥ 9 days post symptom onset) | 89.7% (130/145) (84.7, 94.6) | 99.3% (272/274) (98.3, 100.0) |
|  | | |
| PCR+ controls (n = 145) | N = 32 (Crotty Lab, La Jolla) | |
|  | N = 113 (UNC CP donor cohort) | |
|  | | |
| Negative controls (n = 274) | N = 122 (UNC pre-2019 healthy adults) | |
|  | N = 48 (UNC laboratory archived arboviral samples, TB endemic region) | |
|  | N = 44 (UNC, clinical pre-organ transplant) | |
|  | N = 28 (UNC, clinical HIV+) | |
|  | N = 16 (healthy adults, Crotty Lab, La Jolla) | |
|  | N = 16 (UNC, respiratory illness samples, COVID-19 negative) | |
