# Supplementary material for: Ethnoracial Disparities in SARS-CoV-2 Seroprevalence in a Large Cohort of Individuals in Central North Carolina from April to December 2020
Source: mSphere. 2022 May 19;7(3):e00841-21. doi: 10.1128/msphere.00841-21 (PMC9241523; doi:10.1128/msphere.00841-21)
Supplement: TABLE S6 [file msphere.00841-21-s0007.docx]

| **Table S6. Insurance category by race/ethnicity** | | | | | | | | |
| --- | --- | --- | --- | --- | --- | --- | --- | --- |
|  | **Private** | | **Public** | | **Self-Pay** | | **Other/Unknown** | |
|  | N | (%) | N | (%) | N | (%) | N | (%) |
| **NL White** | 2882 | 37.26 | 4369 | 56.48 | 423 | 5.47 | 61 | 0.79 |
| **NL Black** | 847 | 29.28 | 1791 | 61.91 | 217 | 7.50 | 38 | 1.31 |
| **NL Other** | 480 | 55.43 | 318 | 36.72 | 55 | 6.35 | 13 | 1.50 |
| **Latinx** | 268 | 27.43 | 412 | 42.17 | 237 | 24.26 | 60 | 6.14 |
| **TOTAL** | 4477 | 35.90 | 6890 | 55.25 | 932 | 7.47 | 172 | 1.38 |
