# Supplementary material for: Ethnoracial Disparities in SARS-CoV-2 Seroprevalence in a Large Cohort of Individuals in Central North Carolina from April to December 2020
Source: mSphere. 2022 May 19;7(3):e00841-21. doi: 10.1128/msphere.00841-21 (PMC9241523; doi:10.1128/msphere.00841-21)
Supplement: TABLE S1 [file msphere.00841-21-s0002.docx]

| **Table S1. Hospital Information** | | | | | |
| --- | --- | --- | --- | --- | --- |
| **Hospital Name** | **Bed number** | **ICU bed number** | **Location** | | |
| UNC Medical Center | 950 | 173 | Orange County, NC | | |
| UNC Rex | 660 | 68 | Wake County, NC | | |
| UNC Chatham | 25 | 4 | Chatham County, NC | | |
| UNC Johnston-Smithfield | 199 | 16 | Johnston County, NC | | |
|  | | | | | |
| **Raw sample positivity by hospital** | | | | | |
|  | **4/19-6/20** | **6/21-8/22** | **8/23-10/24** | **10/25-12/26** | **4/19-12/26** |
| Johnston Hospital | 8.10 | 19.45 | 12.69 | 18.62 | 14.21 |
| Chatham Hospital | 6.60 | 10.74 | 13.19 | 10.77 | 9.38 |
| UNC Hospitals | 4.19 | 10.66 | 11.25 | 13.68 | 9.39 |
| Rex Hospital | 4.36 | 6.19 | 8.43 | 13.52 | 8.22 |

**Table S1. Bed numbers and raw antibody test positivity (percent) for each participating hospital.** Number of beds and county location of each hospital in this study^2^.
