# Supplementary figures and images for: Ethnoracial Disparities in SARS-CoV-2 Seroprevalence in a Large Cohort of Individuals in Central North Carolina from April to December 2020
Source: mSphere. 2022 May 19;7(3):e00841-21. doi: 10.1128/msphere.00841-21 (PMC9241523; doi:10.1128/msphere.00841-21)

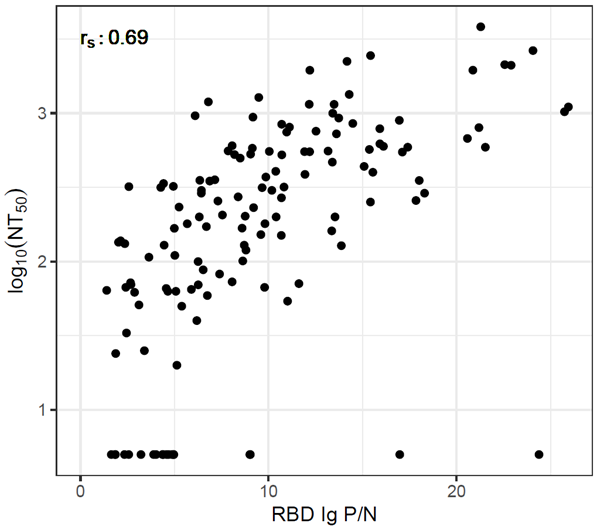

Supplement: FIG S1 [file msphere.00841-21-s0009.tif]
