# Supplementary material for: Ethnoracial Disparities in SARS-CoV-2 Seroprevalence in a Large Cohort of Individuals in Central North Carolina from April to December 2020
Source: mSphere. 2022 May 19;7(3):e00841-21. doi: 10.1128/msphere.00841-21 (PMC9241523; doi:10.1128/msphere.00841-21)
Supplement: TEXT S1 [file msphere.00841-21-s0001.docx]

**Manuscript Title:** Ethnoracial disparities in SARS-CoV-2 seroprevalence in a large cohort of individuals in central North Carolina from April to December 2020

**Supplementary Methods: Bayesian seroprevalence models with unknown sensitivity and specificity**

**1 Determining Test Results**

**1.1 Quantitative test outcome**

To account for plate-to-plate variability (i.e., batch effects), similar to Zhang and colleagues^3^, we used P/N ratios, rather than using the raw optical density (OD) values, defined as

P/N=$\frac{average OD sample}{average OD negative control}$

where the negative control values were those from the same plate as the sample. Accounting for batch effects in the P/N ratio removes the need for defining plate specific cutoffs, and rather we can define one cutoff on how many times larger the sample’s OD value is relative to the corresponding negative control’s OD value.

**1.2 Cutoff Selection**

The CDC recommends selecting a threshold such that the test has 99.5% specificity ^4^. We followed this recommendation here specifying the cutoff to be the standard estimate of the 0.995 quantile (based on the quantile function in R) of the negative lab samples. Using the 274 negative controls, the cutoff was 2.57 with empirical sensitivity of 89.7% and empirical specificity of 99.3%. Therefore, a sample is considered positive if its average OD value is 2.57 or more times larger than the average OD of the corresponding plate negative controls.

**2. Temporal Logistic Model**

We fit a Bayesian autoregressive logistic model to estimate weekly prevalence while accounting for uncertainty in test sensitivity and specificity. Let n_t_ give the number of samples in week t, and y_t_ give the number of samples that tested positive in week t, for t ∈ {1,...,T = 36}. Then

y_t_ ∼ binomial(n_t_,p_t_) t = 1,...,T

were p_t_ gives the probability of a positive test in week t. To account for the error rate of the test, we define

p_t_ =π_t_sens+(1−π_t_)(1−spec)

where π_t_ is the probability an individual has COVID-19 antibodies in week t, sens gives the sensitivity of the test, and spec gives the specificity of the test.

Assuming seroprevalence varies smoothly, we define an AR(1) process for the π_t_ as follows. First, let β_t_ = logit(π_t_). Then we model β_t_ as

β_t_ ∼normal(α+φβ_t−1_,σ_β_2) t=2,...,T

β_1_ ∼ normal(α, 0.5).

As we expect autocorrelation and we are on the logit scale, we expect σ_β_2 to be relatively small, so a relatively vague prior is assumed

σ_β_2 ∼normal+(0,0.5),

where normal+ indicates the folded normal distribution. We found changing the prior variance of σ_β_^2^ had minimal effect on the estimates and associated uncertainty of {π_t_}. Similarly, we put vague priors on α, φ, sens, and spec:

α ∼ logistic(0, 1);

φ ∼ normal(0, 1);

sens ∼ uniform(0, 1);

spec ∼ uniform(0, 1).

Finally, to estimate sensitivity and specificity, we assume

y_spec_ ∼ binomial(n_spec_, spec),

y_sens_ ∼ binomial(n_sens_, sens)

where y_spec_ is the number of negative controls that tested negative out of n_spec_ negative controls. Similarly, y_sens_ is the number of positive controls that tested positive out of n_sens_ positive controls.

**3. Logistic Regression Model**

We fit a Bayesian logistic regression model with main effects for sex, race/ethnicity, age, in/out-patient status, and payor. Interactions were considered, but not found to significantly improve the fit. This model allows us to simultaneously model the hospital data and the lab validation data.

To ensure each category in our main effects had a sufficient sample size, some categories were collapsed. All outpatient, emergency, or unknown patients were listed as “outpatient.” Additionally, the “other” and “unknown” categories for payor were collapsed. Finally, the one patient with sex listed as “X” was removed from the dataset for this analysis.

We define the likelihood

y_i_ ∼ Bernoulli(q_i_) i = 1,...n

where y_i_ is an indicator for whether individual i tests positive for COVID-19 antibodies and q_i_ is the probability of a positive test for individual i. The number of patients is given by n. To account for the error rate of the test, similar to the temporal model, we define

q_i_ =π_i_sens+(1−π_i_)(1−spec)

where π_i_ is the probability individual i has COVID-19 antibodies, sens gives the sensitivity of the test, and spec gives the specificity of the test. Finally, the probability a patient has COVID-19 antibodies is assumed to equal

π_i_ = logit−1 ($\beta_{0t_{i}}$ + **x**′_i_$\boldsymbol{\alpha}_{\boldsymbol{t}_{\boldsymbol{i}}}$)

for the vector of p predictors **x**_i_, coefficients **α**_t_, and intercept $\beta_{0t}$, where t_i_ gives the time period patient i was sampled during, t ∈ {1, . . . , T = 4}. This allows the intercept and coefficients to vary across time periods, but the sensitivity and specificity estimates to be pooled across time. For this analysis, the vector **x** contains indicators for Male, NL Black, NL Other, Latinx, age 18-49, age 50-64, age 65-99, outpatient, public payor, self-pay, and unknown payor. This leaves inpatient, private paying, NL White, females aged 5-17 as the baseline category. Let **x**_i_ be the i^th^ row of the n×p matrix **X**. We calculated the effect of the covariates over the entire study period as

$\bar{\boldsymbol{\alpha}}=\sum_{t=1}^{T} \boldsymbol{\alpha}_{\boldsymbol{t}}/T$,

and present $\exp\left( \bar{\boldsymbol{\alpha}} \right)$ as the average estimated odds ratio.

As before, to estimate sensitivity and specificity, we assume

y_spec_ ∼ binomial(n_spec_, spec)

y_sens_ ∼ binomial(n_sens_, sens)

where y_spec_ is the number of negative controls that tested negative out of n_spec_ negative controls and y_sens_ is the number of positive controls that tested positive out of n_sens_ positive controls.

We chose non-informative priors:

**α**_t_ ∼ normal(**0**_p_, 2**I**_p_)

sens ∼ uniform(0, 1)
spec ∼ uniform(0, 1)

$$\beta_{0t}+\bar{\boldsymbol{x}_{\boldsymbol{t}}^{\boldsymbol{'}}}\boldsymbol{\alpha}_{\boldsymbol{t}}\sim logistic\left( 0,1 \right)\quad t=1,\ldots,T$$

where $\bar{\boldsymbol{x}_{\boldsymbol{t}}}$ is the p-dimensional vector of the average of each column of **X** across the rows corresponding with time period t (i.e. the proportion of observations in each group during that time period). In this way, following Gelman and Carpenter^5^, the prior on β_0_ models the probability of the average patient being seropositive as uniform on the interval (0,1). Note, because the **α_t_** coefficients are on the logit scale, a variance of 2 is relatively vague. For example, this places about 68% probability that an element of **α**_t_ is between -√2 and √2, evaluating to a subpopulation having 0.24 to 4.11 times the seroprevalence of another.

An artifact of the model accounting for uncertainty in test sensitivity and specificity is that when there is low observed positivity, small changes in the estimated specificity can result in large changes in the overall seroprevalence, compared to when there is larger overall positivity. Therefore, there is more uncertainty in these cases (as we observed in earliest time period of our data compared to the latter two). This is because when there are very few positive tests, small declines in the estimated specificity suggest the observed positives should be classified as false positives and the seroprevalence is very low. Without copious amounts of lab validation data, some uncertainty in specificity is expected and this uncertainty will propagate to the seroprevalence and coefficient estimates.

**4. MCMC Algorithm**

The models were fit using a Markov chain Monte Carlo algorithm implemented in Stan^6^. For each model, we ran four chains 5000 iterations each with the first 2500 iterations used as burn-in. The $\hat{R}$ value was within 0.002 of 1 for each parameter, suggesting convergence^7^. The effective sample size was over 2000 for each parameter in the logistic regression model, and over 940 for each parameter in the temporal model.

**5. Quantifying Uncertainty**

For results from the Bayesian models, we reported posterior means and equal-tail 95% credible intervals (i.e., the 2.5% and 97.5% quantiles of the posterior draws). In Table 1, we calculated standard 95% confidence intervals:

$$\hat{p}\pm Z_{\alpha/2}\sqrt{\frac{\hat{p}\left( 1-\hat{p} \right)}{n}}$$

where $\hat{p}$ denotes the observed proportion, $n$ is the sample size, and $Z_{\alpha/2}$ is the $\alpha/2$ quantile of the standard normal distribution.

**6. Demographic data categorization**

To categorize individual clinical encounters associated with the blood draws we sampled, we obtained ICD-10 codes from any inpatient or outpatient visit at the same location within fourteen days of when we received and sampled the blood draw. We prioritized inpatient visits over outpatient visits unless no inpatient visit was available. If there was no visit within the past fourteen days of the blood draw, we instead used the visit closest to the most recent specimen collection date within a thirty-day period. Individuals with no visit at the same location within thirty days of their blood draw were excluded from analysis. To capture any upper respiratory infection, respiratory disease due to external agents, interstitial lung disease, imaging abnormalities of the lung, cough, fever, and dyspnea, we used the International Classification of Diseases, 10th revision (ICD-10) codes J00-J006, J009-J018, J20-J22, J40-J47, J60-J70, J80-J84, J96-J99, R91, R05, R06.0, and R50. COVID-19 diagnosis was defined as presence of the U07.1 code in the visit nearest the sampled blood draw. Likewise, acute or trauma cases were defined as any of the following ICD-10 codes: O00, O01, O02, O03, O04, O07, O08, O015.1, all S codes, all T codes (except T36-T39, T41, T46, T50, T80-T88), all V codes, all W codes, all X codes, all Y codes (except Y62-Y84 and Y-90-99).

Insurance status was determined from the most recent clinical encounter prior to the sampled blood draw. “Private Insurance” was classified as any of the following listed for a patient’s visit: Blue Cross/Blue Shield, Private health insurance, or State Government insurance. “Public Insurance” was classified as any of these following: Medicaid applicant, Medicaid, Medicare, Department of Veteran’s Affairs, Tricare, and Corrections State insurance. “Self-pay” includes anyone paying out of pocket. “Unknown/Other” consists of individuals for whom the health insurance payor was left blank or otherwise unidentifiable, as well as listed insurance that read “Legal Liability / Liability Insurance”, “Other specified but not otherwise classifiable (includes Hospice - Unspecified plan)”, and “Other”.

Race and ethnicity identity was ascertained from that listed in the EMR for each patient. The categories listed under Epic’s EMR that we received included “American Indian or Alaska Native”, “Asian”, “Black or African American”, “Native Hawaiian or other Pacific Islander”, “Other Race”, “Patient Refused”, “Unknown” or “White or Caucasian”. For ethnicity, we received information on whether patients self-identified as “Hispanic or Latino”, or were listed as “Patient Refused” or “Unknown”. In our report, we collapse race and ethnicity from separate variables into a single variable in order to investigate the impact of systemic racism on SARS-COV-2 seroprevalence by both race and ethnicity at the same time, though the constructs of race and ethnicity are inherently surrogate measures of racism and other forms of marginalization^8^.

We therefore binned individuals into the following groups: “Black or African American” that indicated “Non-Hispanic or Latino,” “Patient Refused,” or “Unknown” were binned as “Non-Latinx Black”, similarly for “White or Caucasian” as “Non-Latinx White”, similarly for all other groups as “Non-Latinx Other”. Anyone that indicated “Hispanic or Latino” were binned as “Latinx”, and therefore could self-identify as any of the above race categories. We do not further separate out other intersections of race and ethnicity because the number of individuals becomes too small to make conclusive claims on odds of seropositivity. We here opt to use Latinx in place of “Hispanic” though it is not the only way to refer to this grouping of individuals that often share cultural characteristics, language, religion, and ancestral geography and history^9^. We also compare racial, ethnic, and age demographics in the study population to the demographics of the 6-county area where most of the study population resided using data collected from US Census Data^1^.

**References**

1. Bureau UC. American Community Survey 5-Year Data (2014-2018). <https://www.census.gov/data/developers/data-sets/acs-5year.html>. Published 2019. Accessed May 5th, 2021.

2. Available ICU bed capacity for all NC HSAs, fiscal year 2018. <https://www.pdaconsultants.com/cms/wp-content/uploads/2020/04/Available-ICU-Beds-by-NC-HSA-Maps-and-Data.pdf>. Published 2018. Accessed.

3. Zhang LJ, Zhang JJ, Kubiak RJ, Yang H. Statistical methods and tool for cut point analysis in immunogenicity assays. *J Immunol Methods.* 2013;389(1-2):79-87.

4. Interim Guidelines for COVID-19 Antibody Testing <https://www.cdc.gov/coronavirus/2019-ncov/lab/resources/antibody-tests-guidelines.html>. Published 2020. Accessed.

5. Gelman A, Carpenter B. Bayesian analysis of tests with unknown specificity and sensitivity. *J R Stat Soc C-Appl.* 2020;69(5):1269-1283.

6. RStan: the R interface to Stan. R package version 2.21.1, 2020. <http://mc-stan.org/>. Published 2020. Accessed2020-2021.

7. Gelman A. *Bayesian data analysis.* Third edition. ed. Boca Raton: CRC Press; 2014.

8. Williams DR, Mohammed SA, Leavell J, Collins C. Race, socioeconomic status, and health: Complexities, ongoing challenges, and research opportunities. *Ann Ny Acad Sci.* 2010;1186:69-101.

9. Guidotti-Hernandez NM. Affective communities and millennial desires: Latinx, or why my computer won't recognize Latina/o. *Cult Dyn.* 2017;29(3):141-159.
