# Supplementary material for: Ethnoracial Disparities in SARS-CoV-2 Seroprevalence in a Large Cohort of Individuals in Central North Carolina from April to December 2020
Source: mSphere. 2022 May 19;7(3):e00841-21. doi: 10.1128/msphere.00841-21 (PMC9241523; doi:10.1128/msphere.00841-21)
Supplement: TABLE S7 [file msphere.00841-21-s0008.docx]

| **Table S7. Subset of individuals with recorded Abbott IgG** | | |
| --- | --- | --- |
|  | **Abbott Nucleocapsid ELISA negative** | **Abbott Nucleocapsid ELISA positive** |
| **RBD Ig ELISA negative** | 165 | 2 |
| **RBD Ig ELISA positive** | 9 | 15 |
